# Microfluidic Droplet-Generation Device with Flexible Walls

**DOI:** 10.3390/mi14091770

**Published:** 2023-09-15

**Authors:** Sajad Yazdanparast, Pouya Rezai, Alidad Amirfazli

**Affiliations:** Department of Mechanical Engineering, York University, Toronto, ON M3J 1P3, Canada

**Keywords:** microfluidics droplet generation, co-flow method, flexible walls, droplet-size control

## Abstract

Controlling droplet sizes is one of the most important aspects of droplet generators used in biomedical research, drug discovery, high-throughput screening, and emulsion manufacturing applications. This is usually achieved by using multiple devices that are restricted in their range of generated droplet sizes. In this paper, a co-flow microfluidic droplet-generation device with flexible walls was developed such that the width of the continuous (C)-phase channel around the dispersed (D)-phase droplet-generating needle can be adjusted on demand. This actuation mechanism allowed for the adjustment of the C-phase flow velocity, hence providing modulated viscous forces to manipulate droplet sizes in a single device. Two distinct droplet-generation regimes were observed at low D-phase Weber numbers, i.e., a dripping regime at high- and medium-channel widths and a plug regime at low-channel widths. The effect of channel width on droplet size was investigated in the dripping regime under three modes of constant C-phase flow rate, velocity, and Capillary number. Reducing the channel width at a constant C-phase flow rate had the most pronounced effect on producing smaller droplets. This effect can be attributed to the combined influences of the wall effect and increased C-phase velocity, leading to a greater impact on droplet size due to the intensified viscous force. Droplet sizes in the range of 175–913 µm were generated; this range was ~2.5 times wider than the state of the art, notably using a single microfluidic device. Lastly, an empirical model based on Buckingham’s Pi theorem was developed to predict the size of droplets based on channel width and height as well as the C-phase Capillary and Reynolds numbers.

## 1. Introduction

Microdroplets refer to micro-scale droplets of a target fluid (called a dispersed, or D-phase, fluid) in a suspension fluid (called a continuous, or C-phase, fluid), whose diverse applications have attracted attention [1,2,3]. Microdroplets provide significant advantages in sample processing and monitoring, such as facile automation, increasing practical flexibility, decreasing analysis time, and small volumes. Molecular synthesis [4], diagnostics [5,6], cell biology [7,8], chemical reactions [9], imaging [10,11,12], and food processing and production [13,14] applications have benefited from the use of microdroplets.

Microdroplets can be generally prepared through three main methods: membrane emulsification [15,16], microchannel emulsification [17,18,19], and microfluidic devices [20,21,22]. In membrane emulsification, microdroplets are generated by passing the dispersed-phase target fluid through micron-sized pores of a membrane [15]. Despite the high droplet-generation frequency due to a large number of pores, polydispersity is high (10 to 20% [16]). In the microchannel emulsification method, droplets are generated in the photolithographically prepared microgrooves [17], micro-cutting in stainless steel [18], and PMMA injection molding [19]. The main drawback of this method is generating partially tunable droplets due to its fixed geometry [23]. Devices made by soft lithography, glass capillaries, 3D printing, or their combination are classified as microfluidic devices. Microfluidic droplet-generation techniques have advantages such as the generation of droplets down to 200 nm in diameter [20], production of highly monodispersed droplets [16], size-tunable single and double emulsions [21,22,24], and Janus droplets [25]. 

Two general methods have been developed in microfluidic droplet generation, i.e., active and passive methods. Active methods involve using an external force, while there is no external force in the passive methods. Passive methods are classified into three different categories based on different arrangements and geometries of channels, i.e., the flow-focusing method [26,27,28,29,30,31,32,33], T-junction method [34,35,36,37,38,39,40,41,42,43,44,45], and co-flow method [46,47,48,49,50,51,52]. 

Controlling droplet size is of great importance as it determines droplets’ physical and chemical performance parameters in an application. In the co-flow method, D-phase fluid is injected into the C-phase fluid through parallel respective channels. The advantage of the co-flow method is that it allows the D-phase fluid droplets to be surrounded by the C-phase fluid, isolating the droplets from the channel walls. Therefore, it minimizes the possibility of droplets’ adhesion to the wall [1]. Additionally, the co-flow method’s stable coaxial configuration provides consistency across a broad spectrum of experimental conditions, setting it apart as an effective approach for versatile droplet-size modulation. Droplet sizes ranging from 80 to 553 μm have been generated with various co-flow microfluidic devices [50,53,54,55]. Therefore, the co-flow method was used in this work, which attempts to enhance the capability of this method to produce even wider ranges of microdroplets, but innovatively in a single device.

**Figure 1:**
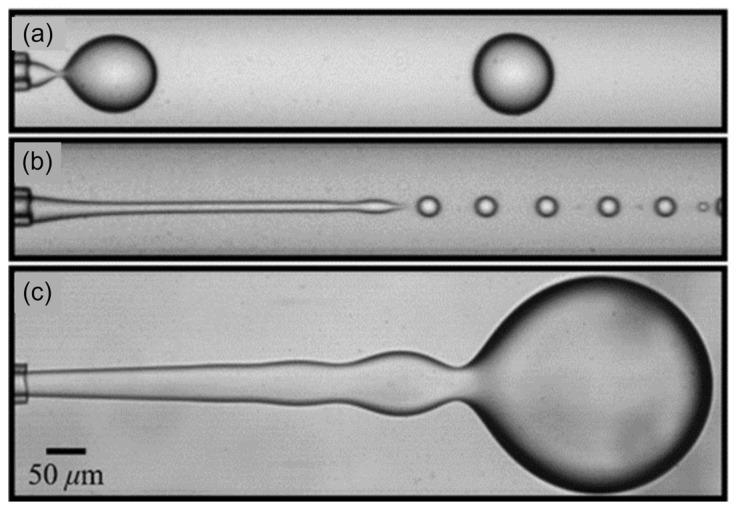
Different droplet-generation regimes in the co-flow method: (**a**) dripping regime, (**b**) narrow jetting regime, and (**c**) wide jetting regime [47]. Reprinted figure with permission from [47], Copyright (2007) by the American Physical Society.

Three regimes are presented in the co-flow droplet-generation method, i.e., dripping (Figure 1a), narrow jetting (Figure 1b), and wide jetting (Figure 1c). In the jetting regimes, there is a jet between the capillary tip and droplet. Polydispersity in the jetting regimes is high because of the perturbations moving along the jet interface [51]. The dripping regime occurs at low C-phase viscous and D-phase inertial forces. This regime has no jet, and the instability is absolute [10]. Perturbations progress downstream and upstream [56,57]. In this kind of instability, perturbations coming from the system give rise to monodispersed droplets in the dripping regime (polydispersity < 3% [58]). For this reason, we attempted to generate droplets in the dripping regime in this paper.

**Figure 2:**
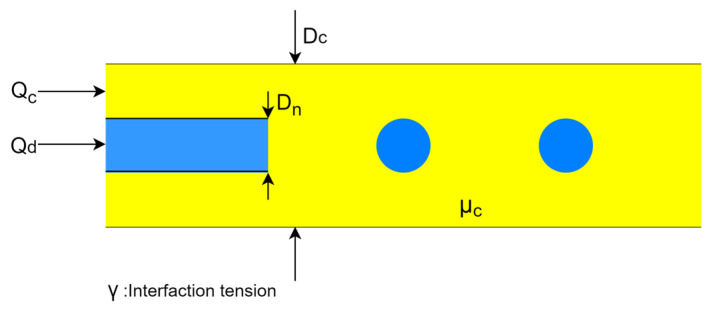
Main parameters affecting droplet size in the co-flow method: C-phase flow rate (Q_c_), C-phase viscosity (μ_c_), interfacial tension (γ), capillary or nozzle tip diameter (D_n_), and channel size (D_c_).

**Figure 3:**
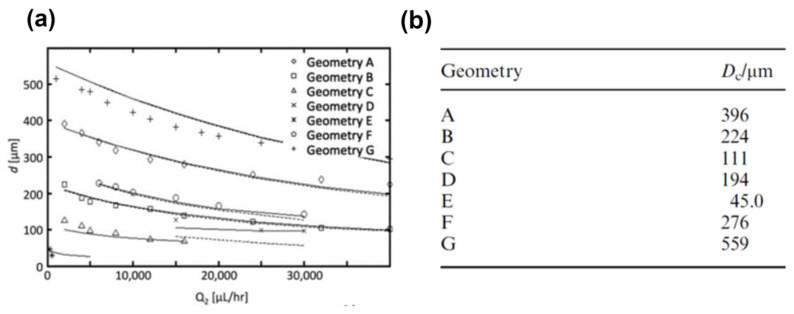
Co-flow droplet generation in devices with various channel sizes [54]. (**a**) Effect of C-phase flow rate on droplet size in eight devices (Geometry A–G), with (**b**) channel sizes ranging from 45 to 559 µm. Reproduced from Ref. [54], with permission from the Royal Society of Chemistry.

Parameters such as the C-phase flow rate (Q_c_), C-phase viscosity (μ_c_), interfacial tension (γ), capillary or nozzle tip diameter (D_n_), and channel size (D_c_) can help control droplet size in the co-flow method (Figure 2). For a given fluid, droplet sizes can hardly be changed continuously by μ_c_ and γ because they are material properties. Also, capillaries in the co-flow systems are generally made of inflexible materials such as glass and 3D-printed materials. C-phase flow-rate-based modulation is applicable only before droplet generation transitions to high-polydispersity regimes [50,53,54]. The only remaining parameter to modulate droplet size is D_c_, which has been demonstrated by using multiple devices with various C-phase channel sizes in Figure 3 [54]. By changing the channel size from 559 to 45 μm in eight devices (geometries A–G), droplet size was controlled from 540 to 50 μm. However, a maximum droplet-size manipulation of approximately 150 µm in a single device was achievable. 

Our design concept in this paper relied on flexible and actuatable C-phase sidewalls to allow for a single device to alter droplet size on demand. We also aimed to provide a comprehensive parametric study on the effect of channel size on device performance, to extend the droplet-size range in a single device, and to provide an empirical equation to predict droplet sizes for such devices.

## 2. Experimental Section

### 2.1. Materials

Polydimethylsiloxane, or PDMS (Sylgard 184 kit, Dow Corning, Midland, MI, USA), pre-polymer was mixed with a 10:1 ratio of base to curing agent. The mixture was then put into the degasification chamber to extract its bubbles. The PDMS mixture was poured on a 3D printed replication mold to fabricate a microchannel for the co-flow droplet-generation process. Flexible silicon tubing (Masterflex, Cole-Parmer Instruments CO, Vernon Hills, IL, USA) was used for the C-phase inlet and the device outlet. A pulled glass capillary (OD: 1.0 mm, 1B100–4, World Precision Instruments, Sarasota, FL, USA) was used for the D-phase inlet. Using this device, droplets of deionized (DI) water were generated using silicone oil (Sigma-Aldrich Co., Darmstadt, Germany) as the D-phase. Properties of materials are given in Table 1.

### 2.2. Experimental Setup

**Figure 4:**
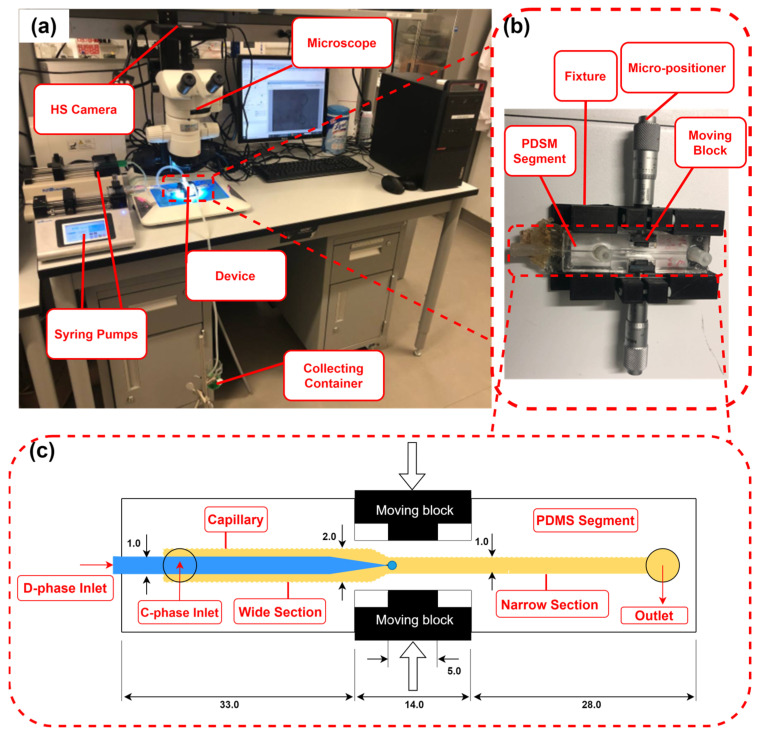
Co-flow droplet-generation device and experimental setup. (**a**) The experimental setup consisted of the developed device, a high-speed camera, a microscope, and two syringe pumps. (**b**) The device comprised a PDMS segment, two glass slides bonded to the top and the bottom of the PDMS segment, a fixture, two micro-positioners, and two moving blocks. The moving blocks were used to squeeze the channel through the micro-positioners. (**c**) A 2D drawing of the PDMS segment with a D-phase glass capillary inserted through a groove into the C-phase channel. Dimensions are in mm.

The experimental setup in Figure 4a consisted of the developed co-flow microfluidic device with flexible walls, a high-speed camera (IL5, Fastec Imaging Corp., San Diego, CA, USA), a C-phase syringe pump (210 Legacy, KD Scientific, Holliston, MA, USA), a D-phase syringe pump (LEGATO 210, KD Scientific, USA), a computer, and a droplet-collecting container. The droplet diameters were measured using ImageJ (NIH, Bethesda, MD, USA). 

### 2.3. Microfluidic Co-Flow Droplet-Generation Device

The co-flow device, shown in Figure 4b, consisted of a PDMS segment with one C-phase inlet and one outlet, a glass capillary D-phase inlet, a fixture, two moving blocks, and two micro-positioners. Figure 4c shows a 2D drawing of the PDMS segment, the glass capillary, and the moving blocks that were used to change the width of the channel. The C-phase channel had two sections: an upstream wide section with a rectangular cross-section of 2 × 1.1 mm, and a downstream narrow section with a rectangular cross-section of 1 × 1 mm. The capillary tip was placed precisely at the mouth of the narrow section. The channel size was tuned on demand by the movement of the blocks orthogonally to the channel using two micro-positioners. Using this mechanism, the channel width was changed from 1 to 0.3 mm around the needle tip.

The soft lithography technique was used to fabricate the PDMS device. PDMS prepolymer was poured into two molds that were 3D printed with a resolution of 32 μm (Connex 3 Object 260 printer, Stratasys Ltd., Waltham, MA, USA). The top and bottom PDMS parts were polymerized on a hotplate at 85 °C for 3 h. The cured PDMS layers were carefully peeled off from the molds and bonded together using an oxygen plasma bonding machine (Harrick Plasma, PDC-001, Ithaca, NY, USA). Then, the PDMS device was bonded to two microscope glass slides from the top and bottom using the oxygen plasma technique. The device was then inserted into the fixture and the micro-positioners were installed on both sides of the fixture (Figure 4b). 

### 2.4. Experimental Procedure

Experiments were conducted at three channel widths (w = 1, 0.65, and 0.3 mm, changed by moving the blocks shown in Figure 4c). The following were the three test modes: (i) constant C-phase flow rate (Q_c_)—the channel width decreased while Q_c_ was kept constant. This mode was used to generate droplets over a wide range because the C-phase velocity and wall effect increased with the decrease in channel width; (ii) constant C-phase velocity (u_c_)—the channel width decreased while u_c_ was kept constant by adjusting the C-phase flow rate. This mode was used to better understand the wall effect on droplet size while keeping u_c_ constant; (iii) constant C-phase Capillary number (${Ca}_{c}=\mu_{c}u_{c}/\gamma_{c}$)—the channel width decreased while Ca_c_ was kept constant. This mode was used to develop an empirical correlation for predicting droplet size in the proposed microfluidic device.

Table 2 shows the levels of parameters in the above three modes under different conditions. The considered parameters were channel width (w), channel height (h), C-phase velocity (u_c_), C-phase flow rate (Q_c_), D-phase flow rate (Q_d_), and C-phase viscosity (μ_c_). The levels of some parameters were physically bounded due to device geometry. The minimum possible level for *w* was 0.3 mm. Also, due to restrictions on squeezing when the channel height was less than 0.7 mm, the minimum level for *h* was 0.7 mm. C-phase viscosity levels were also selected sufficiently high (50–100 cSt) to set lower values for the C-phase velocity. The selected Q_c_, u_c_, Ca_c_, and Q_d_ values were to ensure droplet generation mostly took place in the dripping regime to achieve better monodispersity. 

## 3. Working Principle of the Co-Flow Droplet-Generation Device with Flexible Walls

**Figure 5:**
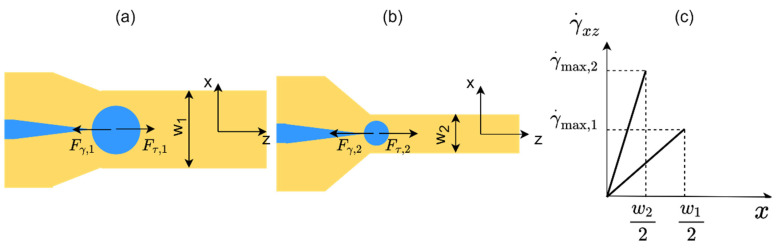
Droplet generation at a constant C-phase velocity, but with two different C-phase channel widths: (**a**) w_1_ and (**b**) w_2_, where w_1_ > w_2_. (**c**) Comparison of shear rates for cases (**a**,**b**). At w = w_2_, the higher maximum shear rate (${\dot{\gamma }}_{max}$) results in the production of smaller droplets.

The operating regime in the droplet generator is dripping. In this regime, the inertial force due to the D-phase flow is negligible. Interfacial tension (F_Υ_) and viscous (F_τ_) forces are dominant and determine the droplet sizes. Figure 5a,b show two droplet-generation cases under a constant C-phase velocity condition. In the first case (Figure 5a), the channel is wide with a width of w_1_, and in the second case (Figure 5b), the channel is narrow with a width of w_2_ (w_1_ > w_2_). Figure 5c shows that the maximum shear rate is higher in the narrow channel based on the axial velocity equation in a rectangular channel ($\dot{\gamma }\sim {\partial u}_{x}/\partial x\;\sim \;w$ [61]). At a higher shear rate, the enhanced viscous force exerted on the droplets leads to the generation of smaller droplets.

## 4. Results and Discussion

### 4.1. Droplet-Generation Regimes and Monodispersity in the Proposed Device

**Figure 6:**
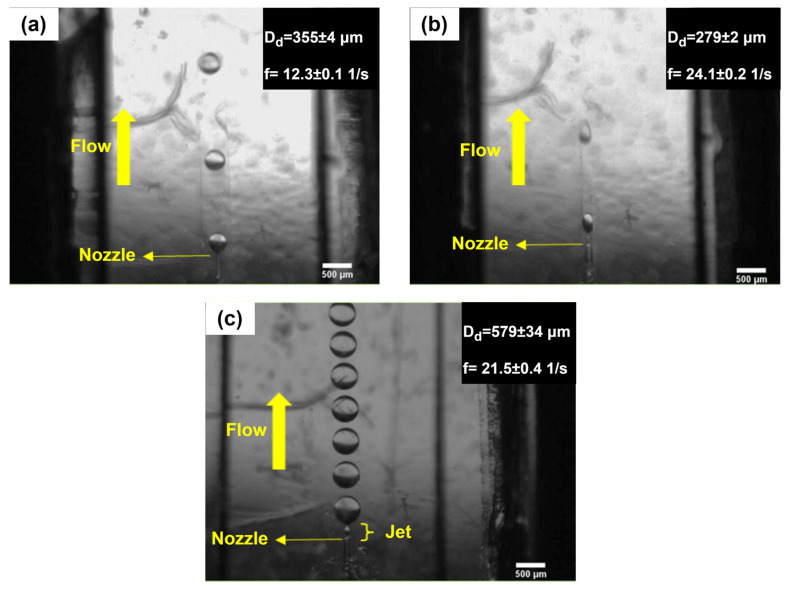
Various droplet-generation regimes in the developed device: (**a**) dripping regime at w = 0.65 mm and (**b**) plug regime at w = 0.3 mm. The conditions for (**a**,**b**) were the same in terms of μ_c_ = 50 cSt, u_c_ = 5 mm/s, h = 0.7 mm, Q_d_ = 1 mL/h, Ca_c_ = 0.007, and We_d_ = 0.0052. (**c**) Transition regime from dripping to wide jetting regime. The conditions were w = 1 mm, μ_c_ = 100 cSt, u_c_ = 4 mm/s, Q_c_ = 10 mL/h, h = 0.7 mm, Q_d_ = 4 mL/h, Ca_c_ = 0.011, and We_d_ = 0.084.

Three distinct regimes were observed in this study: the dripping regime (Figure 6a), the plug regime (Figure 6b), and the transition from dripping regime to the wide jetting regime (Figure 6c). In the plug regime, the thread grew so that it touched the lateral walls while the channel width was at its minimum level. This was the main difference between the dripping and the plug regimes. As the D-phase flow rate increased such that We_d_ ~ O(0.1) [8], the wide jetting regime emerged. In this regime, the inertial force becomes dominant, i.e., a jet is formed, and a droplet is generated at a distance from the capillary tip.

**Figure 7:**
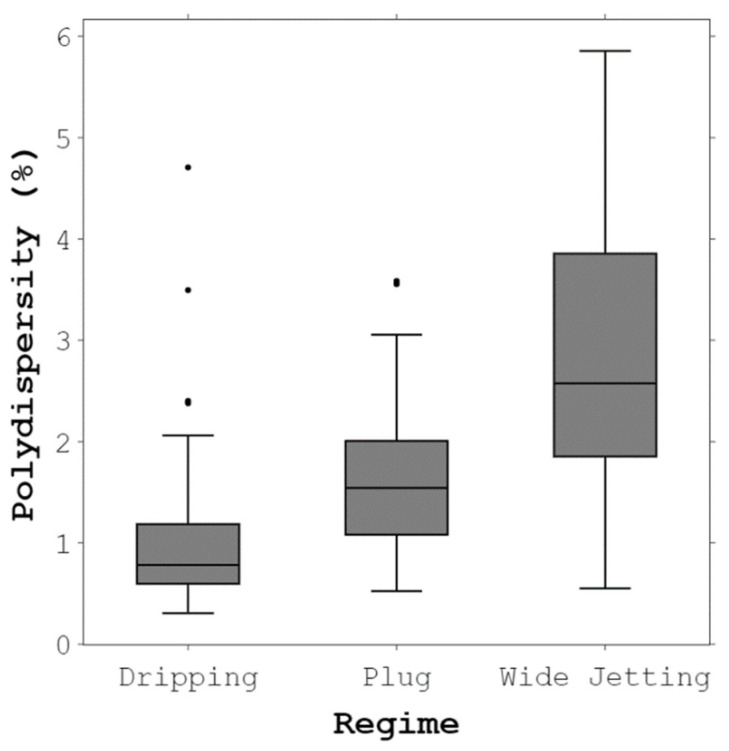
Box plot of polydispersity for the droplet-generation regimes observed in the current study. Each experiment was repeated three times.

Polydispersity is defined as the ratio of the standard deviation of droplet diameter to the mean diameter of the droplet population. Figure 7 shows the box plot of polydispersity for the regimes observed in this study. In the dripping regime, the perturbations from the system occur at a fixed location. Because of this, the median of polydispersity in the dripping regime was less than 1%. However, some outliers may be due to experimental disturbances. The monodispersity of droplets in the dripping regime is consistent with the literature [52,56]. There was a slight increase in the polydispersity of droplets in the plug regime. This increase may result from the instabilities that originated from the walls. Droplets generated in the transition regime to the wide jetting regime were less uniform. The existence of a jet alongside random noises led to a higher polydispersity. Polydispersity is likely to increase with a further increase in We_d_ because the system will operate solely in the wide jetting regime.

### 4.2. Droplet Size at a Constant C-Phase Flow Rate

**Figure 8:**
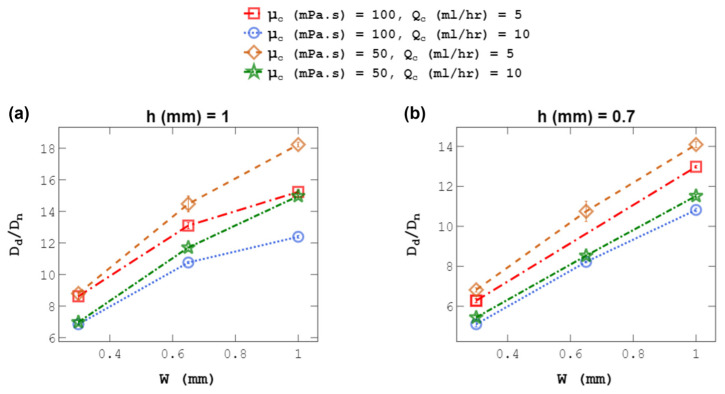
Effect of channel width, w, on droplet size, D_d_, at constant C-phase flow rates, Q_c_, for (**a**) h = 1 mm and (**b**) h = 0.7 mm. Droplet diameters were normalized with the capillary tip size D_n_ = 40 μm. Each experiment was repeated three times.

C-phase flow rate (Q_c_) was kept constant while the channel was squeezed to generate droplets over a wide range. Figure 8 shows the effect of changing the channel width from 1 to 0.3 mm on droplet size using channels with h = 1 and 0.7 mm. As shown, decreasing the channel width substantially reduced droplet diameters, supporting the idea of using channel size though a flexible wall to control droplet size on demand. This method will facilitate the growth of the wall effect as defined above, in addition to an increase in C-phase velocity, giving rise to an increased viscous force causing droplet breakup. Moreover, the results in Figure 8 show that by increasing the C-phase viscosity (µ_c_) and flow rate (Q_c_), at a constant channel width, droplet size decreases due to an increase in the shearing viscous force. 

**Figure 9:**
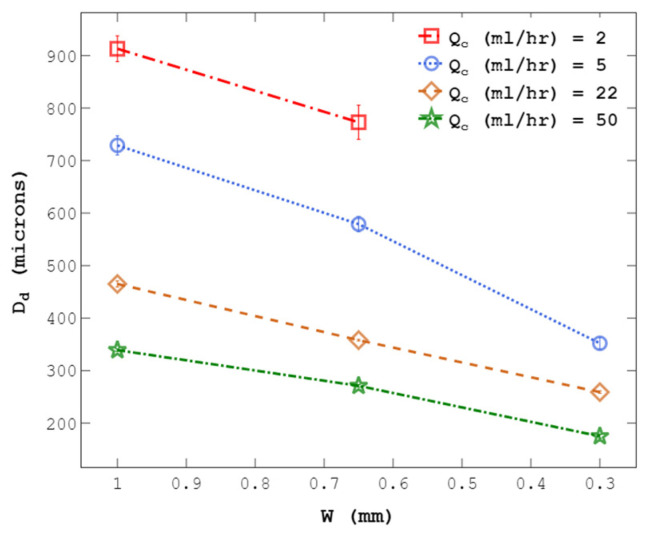
Manipulating droplet size by changing w from 1 to 0.3 mm at four levels of C-phase flow rate (Q_c_ = 2, 5, 22, 50 mL/h). Droplet sizes from 913 to 175 µm were generated (at μ_c_ = 50 cSt and h = 1 mm). Each experiment was repeated three times.

**Figure 10:**
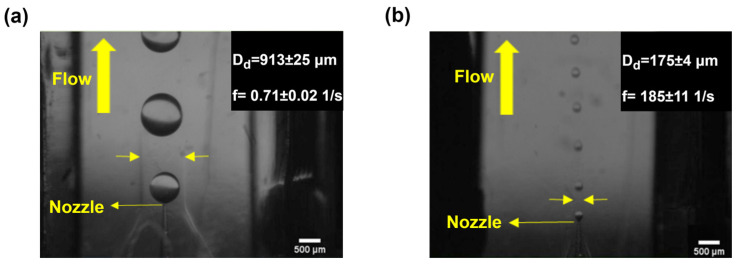
Extending droplet-size range using channel width tuning. (**a**) Channel width of 1 mm resulting in a droplet size of 913 µm at Q_c_ = 2 mL/h. (**b**) Channel width of 0.3 mm yielding a droplet size of 175 µm at Q_c_ = 50 mL/h.

The droplet-size range can be extended when other C-phase flow rate levels are used (Q_c_ = 2, 5, 22, 50 mL/h), as shown in Figure 9. Droplet diameters changed from 913 to 175 μm by tuning the channel width from 1 to 0.3 mm, as shown in Figure 10. 

Table 3 compares the range of droplets generated in this study with previous co-flow devices. The device developed by Chu et al. [61] had the widest range of droplets (298 μm) under the co-flow condition. The current device extended this range by 2.5 times (738 μm). One limitation of the device is that the width cannot decrease below 0.3 mm. In the next generation of devices, the droplet range could be increased even further by employing more flexible walls.

### 4.3. Effect of D-Phase Flow Rate on Droplet Size

**Figure 11:**
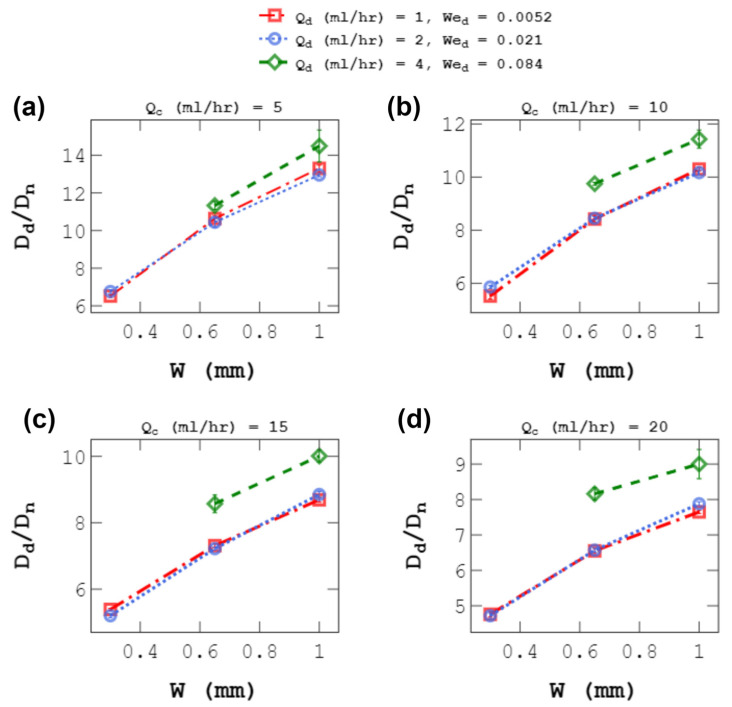
Effect of channel width, w, on droplet size, D_d_, for different D-phase flow rates, Q_d_. Each experiment was repeated three times. Panels (**a**–**d**) represent different Q_c_ values that is given for each plot.

The effect of the D-phase flow rate (Q_d_) on droplet size at a fixed Q_c_ was also investigated. Figure 11 shows the effect of channel width on droplet size for three levels of Q_d_. For all Q_c_ values, when Q_d_ doubled from 1 to 2 mL/h, the maximum change in droplet size was about 5%. This slight change was seen when Q_d_ was lower than 2 mL/h, We_d_ < O(0.1), i.e., in the dripping regime [47]. In the dripping regime, Q_d_ did not significantly alter the force balance between viscous and interfacial tension forces [59]. But when Q_d_ increased to 4 mL/h, droplet sizes increased by 9–24%, depending on the value of Q_c_ and *w*. Also, the average polydispersity increased from 1.3% to 2.9%. The reason for these changes was the transition to the wide jetting regime at Q_d_ = 4 mL/h. The same behavior was observed by Shams et al. [52]. They showed that when We_d_ rose from 0.01 to 0.04, the maximum change in droplet size was about 11%, but when We_d_ increased to 1, the wide jetting regime was formed, and droplet sizes grew by about 34%. The results in this section support previous reports [47,50] on the weak effect of Q_d_ on droplet size.

### 4.4. Droplet Size at a Constant C-Phase Velocity

**Figure 12:**
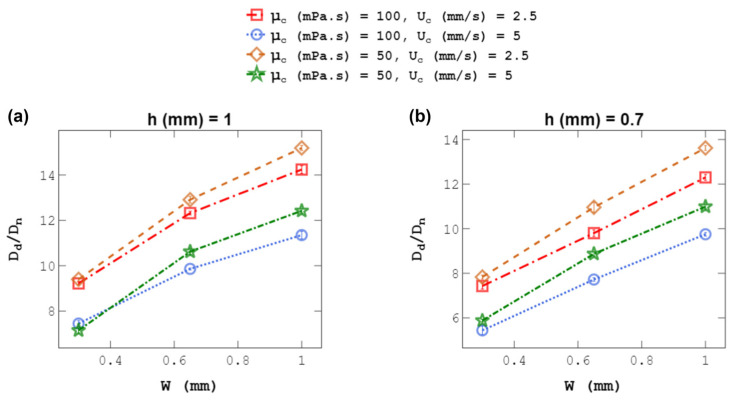
Effect of channel width, w, on droplet size, D_d_, at a constant C-phase velocity, U_c_, for h = 1 and 0.7 mm. Droplet diameters were normalized using capillary tip size D_n_ = 40 μm. Each experiment was repeated three times. Panels (**a**,**b**) are different channel hieghts given on top of each plot.

To further explain the physics behind the observations in the previous sections, the effect of C-phase velocity on droplet size is discussed here. At first glance, the droplet-size decrease in the previous sections may be deemed mainly due to the increase in the average C-phase velocity upon channel-width modulation. However, C-phase velocity is not the only factor, as the wall effect strongly influences droplet size as well. To substantiate our claim, the channel was squeezed at a constant average C-phase velocity. Figure 12 reveals a steady decline in droplet size as channel size decreases. The minimum and maximum droplet-size reduction were 156 μm (at μ_c_ = 100, U_c_ = 5, and h = 1) and 232 μm (at μ_c_ = 50, U_c_ = 2.5, and h = 1) when the channel width was changed from 1 to 0.3 mm. The reduction in the C-phase channel width resulted in an increase in the wall effect and the drag force exerted on the droplet. The intensified drag force surpassed the interfacial tension force more rapidly, resulting in the production of smaller droplets. These findings underscore the intricate interplay of C-phase velocity and wall effects in droplet-generation systems. The variation in C-phase viscosity also played a significant role in droplet generation. Lower C-phase viscosity resulted in a reduction in the viscous force acting on the droplet, leading to a delayed pinch-off and the formation of larger droplets. 

### 4.5. Droplet-Size Prediction Model

Buckingham’s Pi theorem was used to define dimensionless numbers to develop a model for the prediction of droplet diameter for our novel device. Droplet size was assumed to be a function of nine parameters, as shown:

(1) $$D_{d}=f\left(\mu_{c},U_{c},\gamma ,w,h,\rho_{c},U_{d},D_{n},\rho_{d}\right)$$

where $\gamma ,\rho_{c},U_{d},D_{n},\rho_{d}$ denote interfacial tension, C-phase density, D-phase velocity, D-phase nozzle diameter, and D-phase density, respectively. Primary variables are selected to be R_1_ = *U_c_*, R_2_ = γ, and R_3_ = *w*. Based on Buckingham’s Pi theorem, *D_d_* can be a function of 9 − 3 = 6 dimensionless numbers. After determining the dimensionless numbers, dimensionless droplet size (*D_d_*/*w*) can be predicted via these dimensionless numbers:

(2) $$\frac{D_{d}}{w}=f\left(\pi_{1}=\frac{\mu_{c}U_{c}}{\gamma },\pi_{2}=\frac{h}{w},\pi_{3}=\frac{\rho_{c}U_{c}^{2}w}{\gamma },\pi_{4}=\frac{U_{d}}{U_{c}},\pi_{5}=\frac{D_{n}}{w},\pi_{6}=\frac{\rho_{d}U_{c}^{2}w}{\gamma }\right)$$

$\pi_{1}={Ca}_{c}$ and $\pi_{2}=\frac{h}{w}$ were kept as is in Equation (2), but other dimensionless numbers were combined to make them more relevant to the physics of the problem. As a result, droplet size can be explained as a function of C-phase Capillary number (*Ca_c_*), the ratio of the height to the width of the channel (*h*/*w*), C-phase Reynolds number (*Re_c_*), and D-phase Weber number (*We_d_*). ANOVA analysis showed that We_d_ has a weak effect on droplet size and can be neglected (see Supplementary Information online). Therefore, the final form of the correlation can be expressed as:

(3) $$\frac{D_{d}}{w}=f\left({Ca}_{c},\frac{h}{w},{Re}_{c}\right)$$

**Figure 13:**
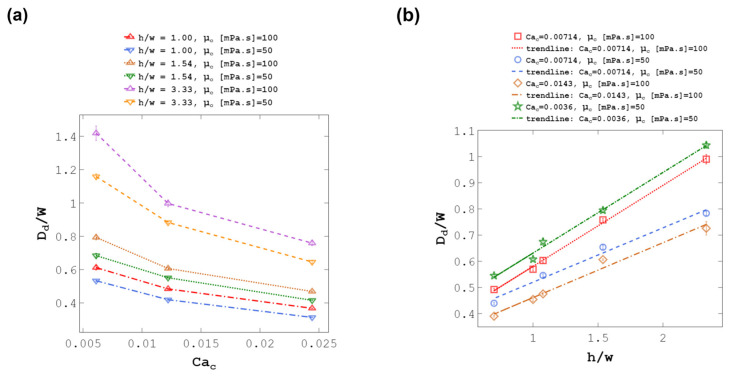
Non-dimensional analysis of the results with the flexible wall co-flow droplet generator. (**a**) Droplet size to channel width ratio, *D_d_*/*w*, versus C-phase Capillary number, *Ca_c_*, at different h/w, and C-phase viscosities, μ_c_. The power function $\frac{D_{d}}{w}\propto {\left({Ca}_{c}\right)}^{A}$ was selected to represent the relation where A is a fitting parameter. (**b**) Droplet size to channel width ratio, *D_d_*/*w*, versus channel height to width ratio, *h*/*w*, at different C-phase Capillary numbers, *Ca_c_*, and C-phase viscosities, μ_c_. There is a linear correlation $\frac{D_{d}}{w}\propto \left(\frac{h}{w}+B\right)$ where B is a fitting parameter Each experiment was repeated three times.

To find the appropriate functional relationship for Equation (3), *D_d_*/*w* is plotted against each dimensionless number (see Figure 13a). Based on Figure 13 and as literature proposes [50], the power function $\frac{D_{d}}{w}\;\alpha {\left({Ca}_{c}\right)}^{A}$ was the best correlation where A is a fitting parameter for the plot shown in Figure 13a. Figure 13b shows *D_d_*/*w* versus *h*/*w* at constant *Ca_c_* and *μ_c_*. As can be seen, a linear function $\frac{D_{d}}{w}\;\alpha \left(\frac{h}{w}+B\right)$ was the best and the simplest equation for the correlation between droplet size and h/w where B is a fitting parameter. For Re_c_, we assumed that the power function can be an appropriate correlation $\frac{D_{d}}{w}\;\propto {\left({Re}_{c}\right)}^{C}$ as it can capture nonlinear trends. Parameter C is a fitting parameter.

**Figure 14:**
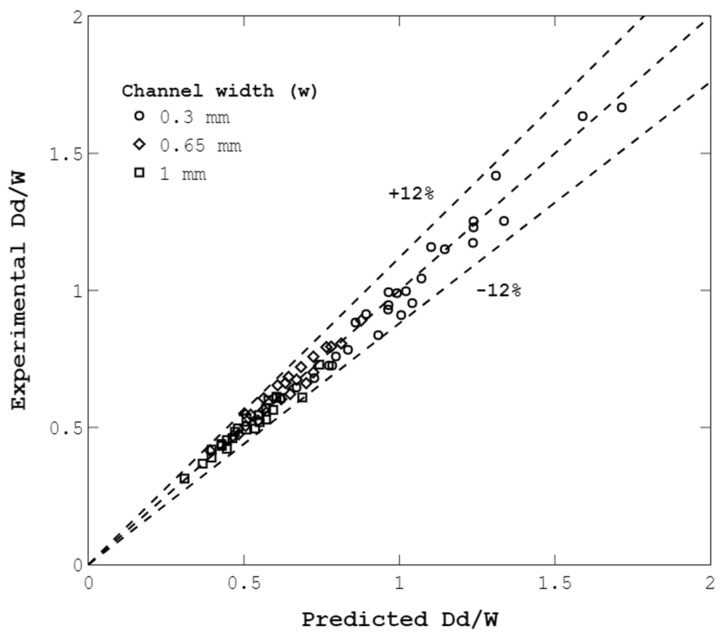
Comparison of predicted and experimental non-dimensional droplet sizes. The maximum error is 11.4%, and the average error is 3.9%.

The final functional form for Equation (3) can be seen in Equation (4). The Fminsearch function of MATLAB software was utilized to find the fitting parameters based on the minimum root mean square error. This function uses the Nelder–Mead simplex algorithm to perform multidimensional unconstrained minimization [62]. Considering the results above, Equation (5) was found and can serve as an empirical model. Figure 14 shows that the proposed equation has an appropriate functional form; thus, the empirical model described by Equation (5) can be used for the prediction of droplet sizes in the proposed device.

(4) $$\frac{D_{d}}{w}=D{Ca}^{A}\left(\frac{h}{w}+B\right){Re}_{c}^{C}$$

(5) $$\frac{D_{d}}{w}=0.0415{Ca}^{-0.235}\left(\frac{h}{w}+1.696\right){Re}_{c}^{-0.125}$$

Further studies with the inclusion of interfacial tension and D-phase viscosity can offer comprehensive insight into the effects of all parameters on droplet size when the channel size changes. One of the limitations of this study was that only two levels of h and µ_c_ were considered. Further research considering wider ranges of these two parameters is recommended to comprehensively explore their effect on the range of droplet sizes.

## 5. Conclusions

In this study, we observed three distinct regimes of droplet formation in a co-flow microfluidic system: dripping, plug, and transition to wide jetting. The dripping regime exhibited high monodispersity, consistent with the literature. As the D-phase flow rate increased, the wide jetting regime emerged, leading to higher polydispersity due to the presence of a jet and random noises.

This study succeeded in showing that the concept of a flexible wall can work to generate a wide range of droplet sizes within the same device. Varying the C-phase flow rate levels allowed for tuning droplet diameters between 913 to 175 μm through channel-width adjustments. The study’s findings indicate the potential of the developed device to extend the droplet-size range by 2.5 times, which is considerably higher than in previous co-flow devices. Future iterations with more flexible walls hold promise for further expanding the droplet-size range and enhancing the versatility of co-flow microfluidic systems for various applications.

Similar to past studies, a weak effect of the D-phase flow rate on droplet size in the dripping regime was seen. Interestingly, the influence of the D-phase flow rate on droplet size in the plug regime was similar to that of the dripping regime due to the similarity of the dominant forces (viscous and interfacial tension forces) in both regimes.

We showed that C-phase velocity is not the only factor determining droplet size. Instead, our findings underscore the prominent influence of the wall effect on droplet size, evident in the consistent reduction in droplet size with decreasing channel width at a constant average C-phase velocity. This effect is attributed to the intensified drag force, which surpasses the interfacial tension force more rapidly, resulting in the production of smaller droplets.

The study also employed Buckingham’s Pi theorem to derive a correlation between droplet size and parameters, such as channel width, C-phase Capillary number, channel height, and C-phase Reynolds number. The derived functional form of the proposed equation demonstrated its suitability, thus validating the empirical model in terms of accurate predictions of intermediate values. Through this comprehensive analysis, we have gained valuable insights into the intricate relationship between droplet size and influencing factors, contributing to a deeper understanding of droplet-generation dynamics in co-flow microfluidic systems. Finally, our novel design eliminates the need to have multiple devices for generating different droplet sizes, and its behaviour is well described by the developed correlations. 

## Figures and Tables

**Table 1:** Physical properties of silicone oils (C-phase) and DI water (D-phase) used in the experiments.

Material	Density ρ (Kg/m^3^)	Viscosity μ (cSt)	Interfacial Tension γ in Water (mN/m)
Silicone oil 1	960	100	35.18 [59] 35.26 [60]
DI water	998	1	
Silicone oil 2	950	50	

**Table 2:** The levels of experimental parameters studied under various droplet-generation modes.

Condition	Q_c_ (mL/h)	u_c_ (mm/s)	${\mathrm{Ca}}_{c}\times$ 10^−3^	w (mm)	Q_d_ (mL/h)	μ_c_ (cSt)	h (mm)
Mode i: Constant C-phase flow rate (Q_c_)	2, 5, 10, 15, 20, 22, 50	16 values between 1.5–11.9, depending on Q_c_ and w	24 values between 2.1–34 depending on Q_c_, w, and μ_c_	0.3, 0.65, 1	1, 2, 4	50, 100	0.7, 1
Mode ii: Constant C-phase velocity (u_c_)	12 values between 2.0–16.75, depending on u_c_ and w	2.5, 5	1.8, 3.6, 7.1, 14.3	0.3, 0.65, 1	1	50, 100	0.7, 1
Mode iii: Constant C-phase Capillary number (Ca_c_)	12 values between 3.4–57.3, depending on Ca_c_ and w	2.1, 4.3, 8.5, 17.1	6.1, 12.2, 24.4	0.3, 0.65, 1	1	50, 100	0.7, 1

**Table 3:** Comparison of the range of droplet sizes generated in this study using a single device with previous co-flow devices. The range of droplets generated in the developed device is 738 µm; it is the widest range compared to previous devices.

D_c_ [μm]	w [μm]	h [μm]	μ_c_ [mPa.s]	D_n_ [μm]	Ca_min_	Ca_max_	D_d_min_ [μm]	D_d_max_ [μm]	D_d_Range_ [μm]	Reference
600	NA	NA	NA	40	NA	NA	255	553	298	Chu et al. (2007) [61]
559	NA	NA	1.2	41	0.00002	0.00096	280	550	270	Erb et al. (2011) [57]
396	NA	NA	1.2	47.4	0.00012	0.0019	223	389	166	Erb et al. (2011) [57]
224	NA	NA	1.2	47	0.00037	0.0060	100	200	100	Erb et al. (2011) [57]
111	NA	NA	1.2	45.4	0.0015	0.0099	80	100	20	Erb et al. (2011) [57]
150	NA	NA	1	40	0.00034	0.010	126	241	115	Perro et al. (2011) [55]
300	NA	NA	49.5	40	0.11	0.51	80	168	166	Deng et al. (2017) [50]
NA	300 to 1000	1000	100	40	0.00085	0.044	175	913	738	This study

## Data Availability

Upon request data may be provided.

## Supplementary Materials

The following supporting information can be downloaded at: https://www.mdpi.com/article/10.3390/mi14091770/s1. Table S1. *P*-values in the ANOVA test showing the significance of the effect of each parameter on the droplet size, *D_d_*. The *p*-value for d-phase Weber number (*We_d_*) is more than 0.05, which shows a weak effect of *We_d_* on *D_d_*.
