# Supplementary material for: Microfluidic Droplet-Generation Device with Flexible Walls
Source: Micromachines (Basel). 2023 Sep 15;14(9):1770. doi: 10.3390/mi14091770 (PMC10536617; doi:10.3390/mi14091770)
Supplement: Supplementary file 1 [file micromachines-14-01770-s001.zip › micromachines-2551416-supplementary.pdf]

micromachines

MDPI

Supplementary Materials

Microfluidic Droplet-Generation Device with Flexible Walls

Sajad Yazdanparast, Pouya Rezai * and Alidad Amirfazli *

Department of Mechanical Engineering, York University, Toronto, ON M3J 1P3, Canada
* Correspondence: prezai@yorku.ca (P.R.); alidad2@yorku.ca (A.A.)

Effect of We_d on the Droplet Size

Analysis of variance (ANOVA) was conducted to confirm if We_d has a significant effect on the droplet size. ANOVA can explore a significant difference between two or more groups. At the presence of a significant difference, the p-value is less than 0.05, and the null hypothesis is rejected. The null hypothesis says that there is no difference among group means. Table S1 shows the p-values of the test. For Ca_c, h/w, and Re_c, p-values were less than 0.05; thus, one can reject the null hypothesis for these parameters. The P-value for We_d was higher than 0.05, implying the non-significant effect of We_d.

Table S1. *P*-values in the ANOVA test showing the significance of the effect of each parameter on the droplet size, D_d. The *p*-value for d-phase Weber number (We_d) is more than 0.05, which shows a weak effect of We_d on D_d.

Dimensionless Numbers	*p*-Value
Ca_c	0.002
h/w	0.047
Re_c	0.006
We_d	0.256
